# Motor Representations and Practice Affect Brain Systems Underlying Imagery: An fMRI Study of Internal Imagery in Novices and Active High Jumpers 

**DOI:** 10.2174/1874440000802010005

**Published:** 2008-01-31

**Authors:** C.-J Olsson, Bert Jonsson, Anne Larsson, Lars Nyberg

**Affiliations:** 1 Department of Integrative Medical Biology Umeå University, S-901 87 Umeå, Sweden; 2 Department of Psychology, Umeå University, S-901 87 Umeå, Sweden; 3 Department of Radiation Sciences Umeå University, S-901 87 Umeå Sweden; 4 Umeå center for Functional Brain Imaging (UFBI)

## Abstract

This study used functional magnetic resonance imaging (fMRI) to investigate differences in brain activity between one group of active high jumpers and one group of high jumping novices (controls) when performing motor imagery of a high jump. It was also investigated how internal imagery training affects neural activity. The results showed that active high jumpers primarily activated motor areas, e.g. pre-motor cortex and cerebellum. Novices activated visual areas, e.g. superior occipital cortex. Imagery training resulted in a reduction of activity in parietal cortex. These results indicate that in order to use an internal perspective during motor imagery of a complex skill, one must have well established motor representations of the skill which then translates into a motor/internal pattern of brain activity. If not, an external perspective will be used and the corresponding brain activation will be a visual/external pattern. Moreover, the findings imply that imagery training reduces the activity in parietal cortex suggesting that imagery is performed more automatic and results in a more efficient motor representation more easily accessed during motor performance.

## MOTOR REPRESENTATIONS AND PRACTICE AFFECT BRAIN SYSTEMS UNDERLYING IMAGERY:

### An fMRI Study of Internal Imagery in Novices and Active High Jumpers

Motor imagery can be defined as mental execution of an action without any muscular movement [1]. Between 70 to 90 % of elite athletes report that they use imagery with the intention of enhancing their physical performance [2], and controlled studies have shown that imagery leads to increased performance on motor tasks (for overview see [3, 4]).

One explanation for why motor imagery can enhance actual motor performance is that motor imagery and motor action engage overlapping brain systems [5, 6]. Components that are critical for motor functions include the primary motor cortex (M1), the pre-motor cortex (PM), and the supplementary motor area (SMA). These regions are also found active in studies comparing motor imagery and motor performance, however, the results vary. Some studies show that there are equal activations in SMA and pre-motor cortex during imagery and physical performance [7, 8]. In other studies SMA and pre-motor cortex are both activated during imagery as well as execution, but during imagery the extent of the activation is smaller [9-2]. Further, in some studies the regional extents are similar, but during imagery the activation is weaker than during physical performance [13-16].

One factor that may account for the weaker and more variable activation patterns during motor imagery is the *imagery perspective* [17]. It is well documented that imagery can be performed using an external or internal perspective. The external perspective is a third-person view, similar to a spectator watching a scene. By contrast, the internal perspective is a first-person view experienced from within. In internal imagery it is typically emphasized that it should feel as if the action is being executed [18]. Based on a review of the literature, Annett [19] concluded that motor imagery may be more similar to action (internal) or perception (external) depending on the imagery perspective.

In order to successfully employ an internal imagery perspective, it should be critical to have well-developed motor representations [5]. In other words, in order for one to “feel as if an action is being executed”, it is crucial to actually have the necessary motor skill for performing the action physically. This view is supported by findings that high ability athletes tend to use internal imagery whereas lower-level athletes use external imagery [20], and predicts that the brain pattern should be a more similar between execution and motor imagery in a skilled relative to a novel state. In an fMRI study, Lacourse *et al.* [21] provided support for this prediction by showing that executed and imagined hand movements were more similar in terms of their functional neuroanatomy in skilled compared to novel learning phases.

In the present study, we compared patterns of brain activity during imagery of a complex motor skill (high-jumping) for two groups. One group was assumed to have well-developed motor representations of the skill (active high jumpers), whereas the other group was assumed not to have well-developed motor representations of the skill (high-jumping novices). To the extent that motor representations influence the imagery perspective and in turn brain activity, we predicted that the high-jumpers would use internal imagery and engage movement-related regions whereas novices would use external imagery and engage visuo-perceptual regions.

A second purpose of this study was to examine whether imagery training within the group of active high-jumpers affected the pattern of brain activity during motor imagery. In a previous study of this sample, (Olsson *et al.* in press), behavioral effects of internal imagery training were evaluated. Half of the active high jumping participants that were used in this fMRI study underwent a training program of internal imagery. The internal imagery training program emphasized critical technical aspects of a high jump, such as the take off and clearing of the bar. The results from the previous study showed that imagery training actually led to improved motor performance. Specifically, the bar-clearance component of the high jump improved after imagery training. One possible explanation for how internal imagery could translate into improved high-jumping performance is through a strengthening of the underlying representation of critical aspects of a jump (e.g. bar clearance). In turn, strengthened representations can then translate into a technically better executed motor action.

If internal imagery strengthens representations, visualization of a high jump should be easier and less demanding for trained participants. Indeed, following motor imagery training, it has been shown that subjects learn to imagine the trained task faster [22]. Such facilitation of imagery could also affect brain activity. Kosslyn *et al.* [23] examined how brain activity differed depending on how long it took to complete an imagery task. They found that there was stronger activity in the parietal cortex when the participants had a longer response time and reduced parietal activity when a task was completed faster. On basis of these previous findings, we predicted that it would be easier to imagine high jumps for the high jumpers that underwent imagery training compared to controls and high jumpers that did not receive any imagery training, and in turn that this ease of imagery would translate into reduced parietal activity.

## METHOD

### Subjects

Twenty four, neurologically healthy, (11 females and 13 males) subjects participated in this study. Twelve of the participants were active high jumpers competing at the national level or higher (mean age 19.3 years, range 18-22 years), and 12 were high jumping novices (control group, mean age 25.1 years, range 21-28 years). The active high jumpers were selected from a national track and field high school. These schools offer a unique opportunity for athletes to attend school and at the same time pursue an elite athletic career. To be selected into these programs you have to be a top level athlete in the nation. The high jumping novices were non-athletic college students. They all reported no background in sports other then the physical education throughout school. Thus, there was a large difference in athletic background and high jumping ability between the high jumpers and controls. The participants had given their informed consent and the study was approved by the ethical committee at the University Hospital of Northern Sweden. Subjects were given a monetary reward ($15) for participating in this study.

### Procedure

The fMRI scanning session was conducted as the second part of a project examining the effects of internal imagery-training on active high jumpers. Therefore, prior to the fMRI session, six of the participants in the group of high jumpers had, in addition to their regular physical practice, been training using internal imagery. At the start of the imagery sessions the high jumpers were instructed, in both verbal and written form, to use an internal perspective. The written instruction explained how to visualize an entire high jump from the start of the runway until the landing. Also, the instruction emphasized critical components such as the take off and clearing of the bar. All through the instruction, an internal perspective was emphasized e.g. *“you feel that the knee…”*. In the oral instruction it was again made sure that the participants understood that it was important to “feel” like the high jump was executed with no muscular movement and not to “see” that the high jump was executed. The other six high jumpers continued with their regular physical practice. To avoid the possibility of experimenter effects for the behavioral measures these six high jumpers also met with the experimenter the same amount of time. The internal imagery training lasted for six weeks with two sessions of imagery each week. During the imagery sessions, the participants were told to imagine high jumps according to the described instruction repeatedly for 1.5 minutes followed by a 30 s rest period. This was repeated four times.

### fMRI Methods

The fMRI scanning was conducted on a Philips Intera 1.5 T system (Philips Medical Systems, Netherlands). Blood-oxygen-level-dependent (BOLD) contrast T2*-weighted images were acquired using a gradient echo-planar imaging (EPI) sequence. The imaging parameters were: echo time: 50 ms, repetition time: 3000 ms, flip angle: 90°, field of view 22 x 22 cm, matrix size: 64 x 64 and slice thickness: 4.4 mm. Thirty-three transaxial slices were collected each 3.0 s, positioned in order to cover the whole brain.

A blocked fMRI design was used. Subjects were asked to visualize according to the internal imagery instruction for 8 s followed by an 8 s rest. This was repeated 20 times. Because of the complex nature of the task (high jump) and to make sure that all participants understood the imagery task, a very clear written and oral instruction of how to make a high jump and how to perform the imagery were given. The task was presented on a semitransparent screen at the end of the bore, using E-prime 1.1 (Psychology Software Tools, PA, USA). The subjects were told to perform the imagery task when an up pointing arrow (↑) was presented and to rest when a plus-sign was presented. Participants were asked not to move and were visually observed during the scanning to make sure that they did not produce any muscular contractions. They were told to only imagine one entire high jump for each 8 s time period and the instruction for the participants emphasized the bar clearance and the take off as particularly important technical details of the high jump. By asking the high jumpers that underwent imagery training approximately how long it took to complete one imagery high jump, it was determined that eight seconds would be appropriate in order for the participants to be able to complete one entire high jump. The participants performed the imagery with their eyes open in order for them to know when to start and stop the imagery. Headphones were used to minimize the noise from the scanner, and cushions inside the head coil helped to minimize head movement. A tilted mirror attached to the head coil was used for the subjects to see the screen. After the scanning none of the participants reported any problems performing the task.

### Statistical Analysis

The fMRI images were first converted to Analyze format using the program MRIcro [24]. Pre-processing of data was done with SPM2 (*Wellcome Department of Cognitive Neurology, London, UK*) and included slice timing correction, realignment, unwarping, normalization to an EPI template in the Montreal Neurological Institute (MNI) space, and finally spatial smoothing (8 mm Gaussian filter). The realignment step gave us further information about possible movements by the subjects. The SPM output reassured us that little movement was made and, thus, no brain activity could be a result of movements by the subjects. Single subject statistical analyses were set up using the general linear model and statistical parametric maps (SPMs) were generated using t-statistics. Random effects analyses were then performed to reveal results on a group basis. An in-house developed software (*DataZ*) was used for visualization. Two sets of statistical analyses were performed:

First, the brain activation during imagery was compared to the rest condition. The threshold was set to p < .001 uncorrected, only showing clusters which had a minimum of 25 voxels activated. This contrast was performed for high jumpers and controls. Possible significant differences in activated regions between the two groups were analyzed using a MANOVA (multivariate analysis of variance) on the peak voxels from each contrast. A significant MANOVA (p < .05) was followed by independent samples t-tests between the two groups for each region. The significance level was set to p < .05. Further, because the group of high jumpers consisted of two sub-groups, one which underwent imagery training and one that did not, individual data were plotted and analyzed using one-way ANOVA for the activated regions to make sure that the jumpers vs. novices group effect was homogenous and not an effect caused by different background of imagery training within the high-jumping groups. Also, possible similarities between high jumpers and controls were evaluated using a conjunction analysis of the imagery-baseline comparison for both groups (p < .001, uncorrected).

The second set of analyses addressed training effects. To test whether internal imagery training modulated activity in the network associated with novice imagery we used the contrast of imagery-baseline for controls as a mask within which we examined training related changes [(imagery-jumpers)]. To test whether training affected regions associated with having a motor representation, the contrast of [(imagery-ontrols)] was used as a mask within which [(imagery-jumpers)] was examined. For the masking analysis, a threshold of p < .01, uncorrected was used.

Regions activated from the masking contrasts were further analyzed using t-statistics to compare the BOLD signal change between the different groups.

## RESULTS

### Brain Activation during Motor Imagery for High Jumpers and Controls

**Fig. (1):**
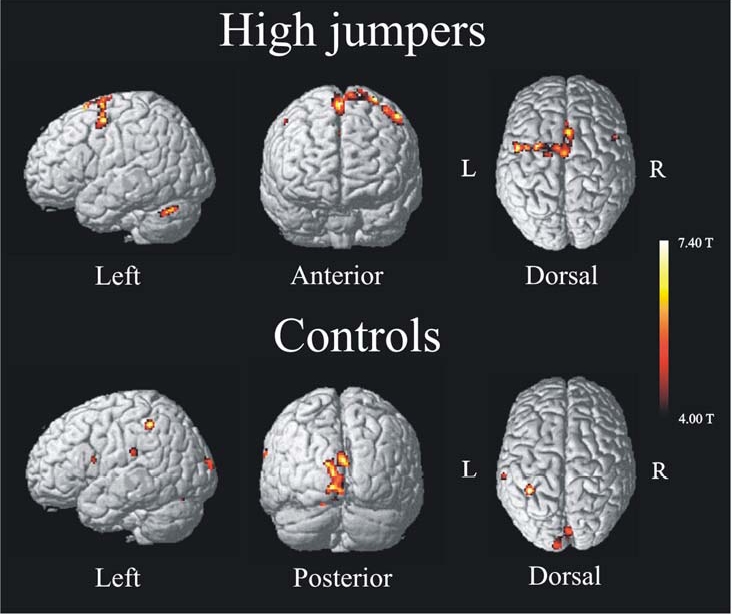
Activation pattern (imagery > baseline) found for high jumpers (top) and controls (bottom) performing the same task, internal imagery of a high jump. High jumpers show motor activation with increased activity in areas such as Pre-motor cortex, SMA and Cerebellum. Novices (controls) show an activation pattern of increased activity in visual and parietal cortex such as superior occipital lobe and inferior parietal cortex. For both groups the significance level was set to p < .001, uncorrected, extent threshold > 25 voxels.

The activation pattern for the high jumpers showed increased activity in several, mainly left-lateralized, motor areas (Fig. **1**, upper). Table **1a** lists brain areas, coordinates (MNI-space), t-values, and spatial extent for regions associated with internal motor imagery of a high jump for the group of high jumpers. The areas found active were SMA, left superior frontal gyrus, left cerebellum, and bilateral pre-motor cortex. To make sure that the left lateralization was not simply a threshold artifact, a paired t-test was made between the BOLD values of all left-sided clusters with the corresponding clusters on the right side, and a significant difference was observed t(11) = 2.9 p < .05. The brain activation pattern for the control group is seen in Fig. (**1a**) (lower). Table **1b** lists areas, coordinates (MNI-space), t-values, and spatial extent associated with internal imagery for the controls. Areas found active were left inferior parietal cortex, superior occipital cortex, left superior temporal cortex, left pre central gyrus, and right lingual gyrus. The SMA was also found active for the controls but at a lower extent threshold (x, y, z = 4, 8, 56, k = 23).

Individual data from the differentially activated regions for the high jumpers are presented in Fig. (**2**). One-way ANOVAs did not reveal any differences as a function of imagery training (all F’s < 1.6; all p’s >.05).

**Fig. (3):**
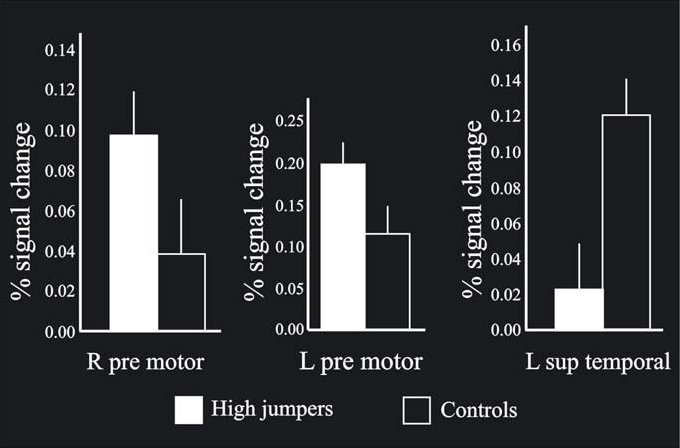
When comparing the BOLD signal change between the groups on the local maxima (p < .05), bilateral pre-motor cortex was found significantly more active for high jumpers compared to the controls, with the left side stronger than the right side. Also, left superior tempo-ral cortex was significantly more activated for the controls compared to the high jumpers. In addition, the bars shown in the Figure indicate standard errors.

The MANOVA across groups (high-jumpers vs controls) for the BOLD values from the highest local maxima (Table **1**) was significant F(1, 22) = 2.71, p < .05. Further analysis of the BOLD-values revealed a significant difference in three regions (Fig. **3**). For two local maxima, the high jumpers had significantly stronger BOLD signal change compared to the controls. These regions were left pre-motor cortex [(x, y, z = -50 -2 54), t(22) = 1.95], and right pre-motor cortex [(x, y, z = 52 10 52 ), t(22) = 1.70]. For one local maximum, the controls had significantly stronger BOLD signal change. This was in left superior temporal gyrus [(x, y, z = -64 -28 24) t(22) = 2.9].

These findings highlight pronounced differences between skilled jumpers and novices during motor imagery. This was further underscored by the results of a conjunction analysis of the overlap in activation patterns for the two groups. This analysis revealed minimal commonalities. The only region where significant overlap was found was the SMA [(x, y, z = -4 -4 76), t = 4.18; k = 3 voxels].

### Brain Activation Associated with Imagery Training

The first masking contrast, where the novices were used as a mask, revealed a local maxima in the left posterior parietal cortex, see Fig. (**4**) (BA 40, x, y, z = -62 -34 42). This area was more activated for high jumpers that did not undergo imagery training and for controls relative to imagery trained jumpers. Analysis of the BOLD signal change for the local maxima in parietal cortex revealed a significant difference between the imagery-trained high jumpers and the non-imagery trained high jumpers, [t (10) = 1.83]. There was also a tendency to a significant difference between imagery trained high jumpers and controls [t(16) = 1.51, p = .075]. The second masking, in which the high jumpers were used as a mask, did not reveal any significant effects.

## DISCUSSION

The purpose of this study was to evaluate similarities and differences in neural activity during imagery of a complex motor skill (high jump) for novices and persons having well developed motor representations of the skill. There were pronounced differences in brain activation between groups. The high jumpers showed increased activity in motor regions such as SMA, pre-motor cortex and cerebellum. This was true regardless of whether they had received imagery training or not. Activation for the controls was found in visual and parietal regions such as occipital cortex and inferior parietal cortex. Further analysis of the BOLD-values for these regions revealed that the high jumpers had significantly stronger activation in two areas (bilateral pre-motor cortex), and that the controls had significantly stronger activation in one area (left superior temporal gyrus). Roland *et al.* [25] argued that SMA is responsible for internally guided actions, both executed and imagined. This view is consistent with our finding, at least in terms of imagined actions, that both groups had SMA activation, although weaker for the novices. This finding of a general role of the SMA in imagery of high jumping is in keeping with the results from a study by Owen *et al.* [26] where it was found that SMA was consistently active during tennis imagery.

### Motor Representations

A main hypothesis was that high jumpers have more developed motor representations of a high jump which in turn facilitate motor activation during motor imagery. A possible reason why imagery enhances motor performance is because that motor imagery and motor action engage overlapping brain systems [5]. If there is a motor representation established, such as it is assumed for the high jumpers, there will be an increase of motor activity during motor imagery, leading to a strengthening of the neural pathway for the motor task and subsequently an improved actual motor performance. Hence, the results of the present study argue for a functional equivalence between execution and imagery for skilled actors. The left hemispheric dominance (Fig. **1**, top) for the high jumpers when performing the imagery task is reasonable. Other studies of imagery have reached similar findings [27, 28]. In studies of bimanual tasks Johansson *et al.* [29] explained left-lateralization of activity in pre-motor areas to be critical for executing sensory-motor programs irrespective of acting hand. Thus, the left hemisphere has a prominent role for motor execution. To the extent that motor imagery may be seen as a preparation for execution, the results from this study are sensible and suggest a left lateralization of brain activation during execution of imagery on the basis of established motor representations.

There was a significant difference in bilateral pre-motor cortex where the high jumpers had stronger activation in regions important for execution and storage of motor representations compared to the controls. High jump is a complex motor skill and Malouin *et al.* [30] discovered that an increase of the complexity of the imagined movement increased the activation in the pre-motor cortex. One possible interpretation is that the pre-motor cortex stores the motor representation of the high jump. Several studies support this line of reasoning. Meister *et al.* [31] found that the pre-motor cortex is related to long term practice of motor tasks. Sakai *et al.* [32] developed a model for motor control which suggests that information converge in the pre-motor cortex to generate a final motor program, and also that the pre-motor cortex encodes detailed action plans for complex movements [33]. Gaser and Schlaug [34] found a positive correlation between gray matter volume in pre-motor cortex with increased musician status. Thus, the pre-motor cortex seems important for the planning and execution of complex motor performance [35]. Pre-motor cortex also appears to reflect the association between sensory cues and motor commands [36, 37]. In studies of primates, neurons in the ventral part of pre-motor cortex fire when natural actions are performed, such as reaching, and also during observation of someone else performing the same familiar action [38, 39]. Our results, together with the above described studies, suggest that activation in pre-motor cortex during motor imagery requires an already established motor representation.

By contrast, for novices, motor imagery of a complex skill will not generate the same neural pattern as real action. Although given the same task, with the same instructions, both verbally and written, with the internal perspective emphasized there were significant differences of activity between the controls and the high jumpers. Instead of having motor activity, the controls showed a visual activation pattern. We hypothesize that the participants created an image watching a high jump instead of feeling it. Thus, the controls used an external perspective and therefore recruited a visual activation pattern. This view is supported by a study showing that visual regions of the brain are activated during visual imagery of motor actions [12]. Also, other studies have shown that visual imagery share common neural networks with visual perception and those regions of the visual cortex are activated during visual imagery tasks [40, 41]. The imagery task required participants to construct an image in which they were taking part in an event that they had never previously experienced. When studying the neural substrates of envisioning the future Szpunar *et al.* [42] showed that future and past events share similar neural networks, and that one would base the future images on representations from the past. In the present study, only the high jumpers could integrate the imagery with a representation from the past. The controls had to create a new image because they did not have the motor representation to start with. Therefore, we propose that the reason why the controls were not able to have a motor activation pattern was because they did not have the proper motor representations.

In further support of the notion that the controls used an external perspective was that in one peak the controls had significantly more activation then the high jumpers. This peak was located in the left superior temporal cortex, just on the boundary to the inferior parietal cortex, and the cluster of activation expanded into this area of the brain. Such a temporo-parietal activation pattern could also reflect that the novices had problems using the first person perspective and instead used a third person perspective. Despite instructed to feel the action, the participants may have repeated the instruction verbally in their mind and therefore engaged in a task more similar to auditory imagery. In fact, similar regions have been found activated in studies of musicians playing music in their minds [43, 44], and also in studies of auditory imagery [45] underpinning our proposal.

### The Effects of Imagery Training

**Fig. (4):**
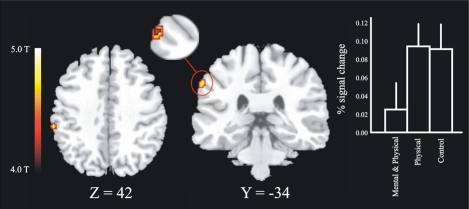
The masking contrast revealed an area (28 voxels) in posterior parietal cortex within which a three voxel large overlap between the controls and the non-imagery trained high jumpers was found. The slice figure show the regions for the inclusion mask in horizontal and transversal direction, in which the overlap was found (enlarged picture). Also shown are the BOLD signal changes for the three different groups on this peak (x y z = -62 -34 42). There was a significant difference (p < .05) between the group of high jumpers that did train imagery (Mental & Physical) and the group of high jumpers that did not train imagery (Physical).

A second purpose of this study was to evaluate the effects of imagery training on patterns of brain activity for participants with well-established motor representations of a high jump. After 6 weeks of imagery training, it was found that brain activity in posterior parietal cortex (Fig. **4**) was lower for high jumpers that had been training imagery compared to the high jumpers that did not take part in imagery training as well as the high-jumping novices. In keeping with previous studies, we hypothesize that imagery training strengthened mental representations of at least some high-jumping components [5].

The parietal cortex has been associated with mental imagery [46], and with evaluation of both real and imagined motor performance [47]. Stronger representations should make the imagery task less cognitively demanding, thereby leading to reduced parietal activity [23]. Stronger mental representations may also underlie the facilitation of motor behavior following imagery training that was observed in our previous study (Olsson *et al.* in press). There is evidence that the parietal cortex is implicated in the formation of motor intentions [48] and when converting sensory information into motor commands [49], Also, the parietal cortex could be a site for the storage of representations of some high-jumping components, notably spatial information (cf., [50]). A reduction of activity in the parietal cortex after training is in line with demonstrations for other kinds of representations that neural activity decreases as a function of repeated stimulus presentation and task execution [51-53].

Taken together, our findings indicate that imagery training results in stronger representations of the complex actions described in the training program, which in turn facilitates imagining of and actually carrying out a high jump.

### Practical Implications

The results from this study indicate that it is important to understand how different the brain behaves during imagery depending on the level of experience of a particular skill. Also, studies examining the use of imagery among athletes show that athletes tend to use imagery just prior to competition to a greater extent than during training [2]. However, the present study suggests that athletes and coaches should be aware of potential differences between using imagery occasionally relative to more consistently. If only used prior to competition, the activation pattern would not solely be motor it would be cognitive in terms of parietal activation as well. A possible effect could then be that the athlete may have difficulties to integrate the details of the imagery into the real action. This may then result in that the athlete will not get the intended benefits of the imagery. Consequently, this study could affect how frequently one engages in imagery training.

Beyond the sports domain, our findings have implications for rehabilitation after an accident or injury. It has been proposed that imagery may be effective in rehabilitation of movement disorders [54, 55]. However, if the use of motor imagery to improve performance rests on the assumption of functional equivalence between performing the action and imagining the action, it is important to make sure that motor representations exist (i.e. that familiar actions are imagined, cf., [26]). If not, a different (external) mental strategy will likely be applied and possibly limit the results of the imagery training.

## CONCLUSIONS

The results from this study suggest that in order to access motor regions, especially the pre-motor cortex, during motor imagery, a motor representation must be established. Lacourse [21] predicted that imagery would be efficacious in both novel and skilled phase of motor learning. However, even though we did not study the neural activation during an actual high jump, the results from this study suggest that the functional equivalence between execution and imagery depends on the level of experience and that the differences between imagery of a novel and a skilled phase of a motor action therefore are more profound. Thus, strengthening of a motor pattern for a specific action is therefore more likely to occur when a certain level of skill has been established.

## Figures and Tables

**Fig. (2):**
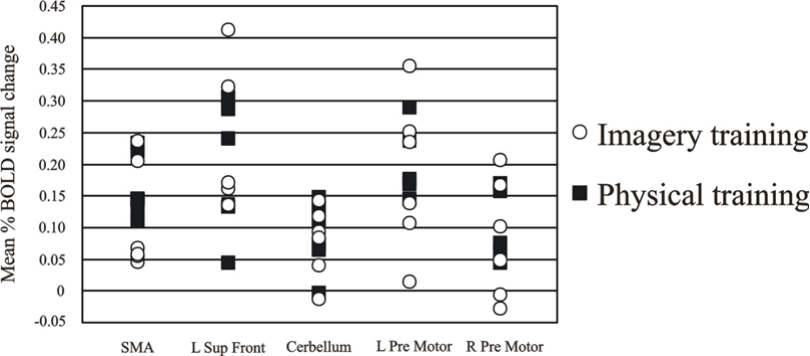
Individual data over the regions activated for the active high jumpers compared to baseline rest. Despite the different background in imagery training the group is homogeneous and therefore we can conclude that the group does not consist of two sub-groups.

**Table 1.** Local Maxima of Regions Activated during Internal Imagery of a High Jump Compared to the Resting Condition

	Anatomical Region	BA	X	Y	Z	t	Extent
**1a** **High jumpers**							
** **	SMA	6	2	14	66	7.51	642*
** **			-2	-6	68	6.24	
** **			-2	0	60	4.94	
** **	Left superior frontal	6	-30	0	70	7.34	*
** **	Cerebellum		-34	-64	-30	7.23	141
** **	Left pre motor	6	-50	-2	54	7.20	*
** **			-40	-2	60	6.11	
** **			-16	-10	78	5.09	
** **	Right pre motor	6	52	10	52	5.92	27
**1b** **Controls**							
** **	Left inferior parietal	40	-38	-44	50	6.39	91
** **	Superior Occipital	18	-8	-90	-6	5.40	531
** **		18	-6	-90	8	5.29	
** **		18	4	-86	18	5.25	
** **		17	-12	-100	16	5.17	
** **	Right lingual	18	14	-70	-4	5.20	93
** **		18	14	-80	-6	4.95	
** **	Left Superior temporal	48	-64	-28	24	4.96	28
** **	Left pre motor	6	-52	8	16	4.75	29

^*^these local maxima were found in the same cluster.
